# Emerging Synaptic Molecules as Candidates in the Etiology of Neurological Disorders

**DOI:** 10.1155/2017/8081758

**Published:** 2017-02-26

**Authors:** Viviana I. Torres, Daniela Vallejo, Nibaldo C. Inestrosa

**Affiliations:** ^1^Centro de Envejecimiento y Regeneración (CARE), Departamento de Biología Celular y Molecular, Facultad de Ciencias Biológicas, Pontificia Universidad Católica de Chile, Santiago, Chile; ^2^Centre for Healthy Brain Ageing, School of Psychiatry, Faculty of Medicine, University of New South Wales, Sydney, NSW, Australia; ^3^Centro de Excelencia en Biomedicina de Magallanes (CEBIMA), Universidad de Magallanes, Punta Arenas, Chile

## Abstract

Synapses are complex structures that allow communication between neurons in the central nervous system. Studies conducted in vertebrate and invertebrate models have contributed to the knowledge of the function of synaptic proteins. The functional synapse requires numerous protein complexes with specialized functions that are regulated in space and time to allow synaptic plasticity. However, their interplay during neuronal development, learning, and memory is poorly understood. Accumulating evidence links synapse proteins to neurodevelopmental, neuropsychiatric, and neurodegenerative diseases. In this review, we describe the way in which several proteins that participate in cell adhesion, scaffolding, exocytosis, and neurotransmitter reception from presynaptic and postsynaptic compartments, mainly from excitatory synapses, have been associated with several synaptopathies, and we relate their functions to the disease phenotype.

## 1. Introduction

**Figure 1:**
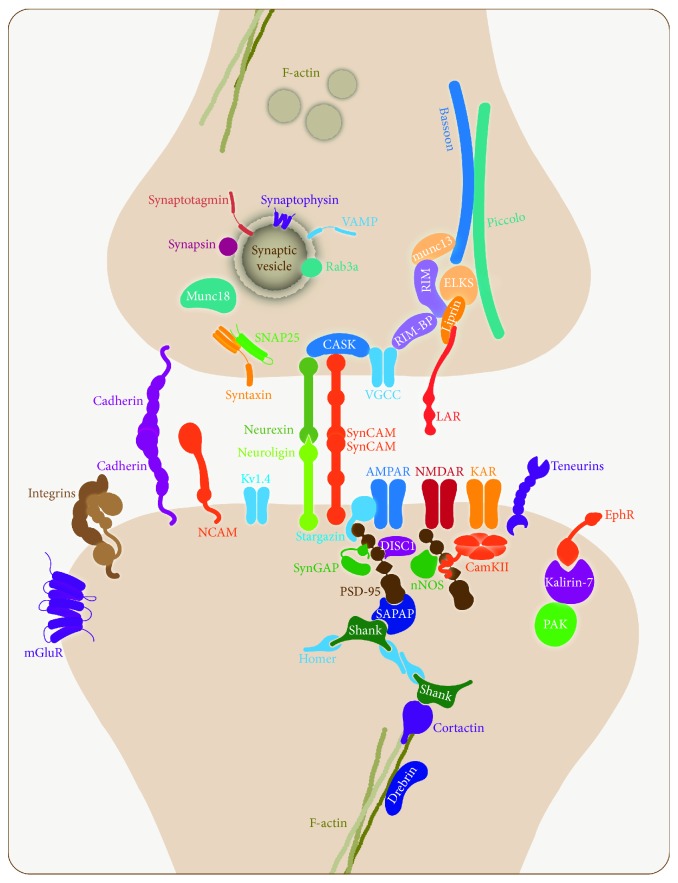
Molecular composition of a central chemical synapse. The image shows a typical excitatory synapse in the CNS. Pre- and postsynaptic proteins are organized in macromolecular functional complexes playing different roles in scaffolding, exocytosis, endocytosis, and signaling in their respective compartments. In addition, the most relevant adhesion molecules are represented.

Communication among neurons in the central nervous system (CNS) is mediated by specialized contacts named synapses that are formed by presynaptic and postsynaptic compartments. The presynapse contains the active zone (AZ), a region that concentrates proteins involved in the recruitment and fusion of synaptic vesicles (SVs), which release neurotransmitter into the synaptic cleft [1, 2] (Figure 1). The postsynaptic side contains the postsynaptic density (PSD) containing the receptors and the signaling machinery that respond to the presynaptically released neurotransmitter, propagating neuronal communication through an action potential [3] (Figure 1). Synapses form during CNS development in a space and time-dependent manner, and these structures are very dynamic in the adult, exhibiting plasticity in response to prevailing physiological requirements.

In the previous three decades, the molecular composition and the organization of the pre- and postsynaptic compartments have been greatly elucidated by a combination of biochemistry, proteomic, genetic, superresolution microscopy, and 3D electron microscopy techniques [4, 5]. Furthermore, interactors with most of the synaptic proteins have been identified, allowing the construction of an intricate protein network. Despite the latter, to translate this protein network into synapse function and efficacy is a complex task because some protein-protein interactions are more stable while others are temporal in response to plasticity events [6–8]. Furthermore, some proteins have diverse isoforms with a spatial-temporal expression pattern that sometimes partially overlaps. The abnormal expression of a synaptic protein and/or mutations and consequent perturbations in synapse physiology might produce aberrant neuronal circuits, synaptic dysfunction, and finally the development of a neurological disease [9–11].

**Figure 2:**
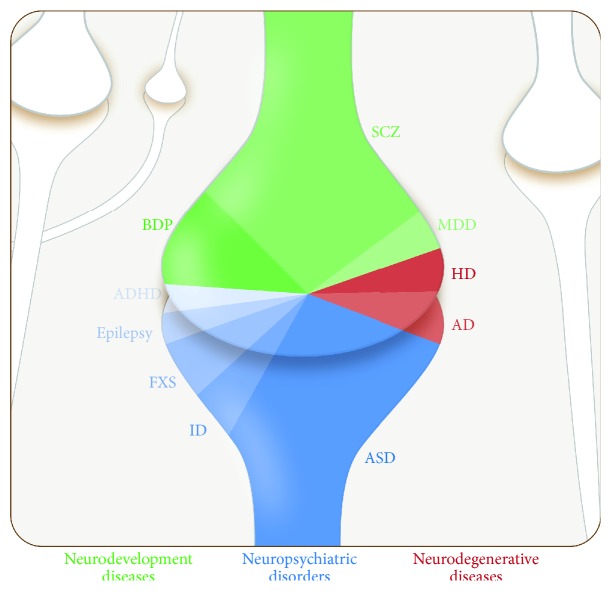
Schematic representation of neurological disorders associated with synaptic protein dysfunction. The image summarizes the neurological diseases described in this review represented by color code: neurodevelopmental (green spectrum), neuropsychiatric (blue spectrum), and neurodegenerative (red spectrum). The number of synaptic proteins involved in each category is proportionally illustrated. AD, Alzheimer's disease; ADHD, attention deficit hyperactivity disorder; ASD, autism spectrum disorder; BPD, bipolar spectrum disorder; FXS, Fragile X syndrome; HD, Huntington's Disease; ID, intellectual disability; MDD, major depressive disorder; SCZ, schizophrenia.

Human genetic studies and animal models of neurological diseases have led to an emerging concept in neurobiology; the term is “synaptopathy,” which refers to brain disorders that have arisen from synaptic dysfunction, including neurodevelopmental (autism spectrum disorders (ASD), intellectual disability (ID), Fragile X syndrome (FXS), Down Syndrome, attention deficit hyperactivity disorder (ADHD), and epilepsy) and neuropsychiatric disorders (bipolar disorder (BPD), schizophrenia (SCZ), and major depressive disorder (MDD)) and neurodegenerative diseases (Alzheimer's disease (AD), Huntington's Disease (HD), and Parkinson's Disease) (Figure 2).

Among the neurodevelopmental disorders, ASD and FXS are synaptopathy-related diseases that are mostly determined by genetic factors. On the one hand, ASD is heritable in 80% of cases, and impaired individuals manifest a variety of intellectual deficiencies from social communication deficits to repetitive and abnormal behaviors [12]. On the other hand, patients with FXS, which is the most common form of inherited mental retardation caused by transcriptional silencing of the fragile X mental retardation protein (FMRP), display ASD-associated symptoms such as ID, altered social interactions, and delayed speech [13]. Regarding neuropsychiatric disorders, SCZ and BPD are strongly linked to genetic and environmental factors. SCZ patients develop abnormal social behavior together with false beliefs, anxiety disorders, and confused thinking, symptoms that are pathophysiologically triggered by synaptic dysfunction resulting from a reduction in the dendritic spine density [14]. Patients affected by BPD manifest periods of depression or elevated mood associated with psychotic attacks that are often related to an elevated risk of self-harm or suicide [15]. In reference to neurodegenerative diseases, the pathology of AD is characterized by the accumulation of senile plaques in the brain, which result in the abnormal amyloid-*β* (A*β*) peptide processing of the amyloid precursor protein (APP), neurofibrillary tangles as a consequence of tau hyperphosphorylation, synaptic disruption, and selective neuronal loss in brain areas associated with memory and cognition. Hence, AD is considered the most prevalent neurodegenerative disease in the elderly population and the most common form of dementia [16]. HD is also a progressive neurodegenerative disorder with symptoms that include cognitive disturbances, mood disorders, and motor abnormalities caused by a mutation in the huntingtin (Htt) protein [17]. Therefore, it is evident that a unique impairment in a single component of this convoluted system, that is, the synapse, can compromise proper synapse function and result in synaptopathy. Understanding the molecular mechanisms leading to synaptic dysfunction will contribute to the development of suitable synapse-targeted therapies for neurodevelopmental, neuropsychiatric, and neurodegenerative disorders.

Here, we describe pre- and postsynaptic proteins that are involved in the pathology of neurological disease originating at chemical synapses in the CNS and are known to support synaptic function via different mechanisms, including adhesion, scaffolding, SV cycling, and signaling.

## 2. Presynaptic Proteins

Presynaptic sites are characterized using electron microscopy by an electrodense material that represents the AZ where specific proteins aggregate to regulate the cycle of SVs. The AZ translates an action potential into a chemical signal that induces the release of neurotransmitters into the synaptic cleft. Synaptic vesicles undergo cycles of exocytosis and endocytosis regulated by AZ proteins. AZ proteins participate in the active modulation of exocytosis according to the circuit requirements. In fact, once synapses are established, AZ undergoes molecular remodeling during their lifespan to support the requirements of synaptic activity and plasticity. Therefore, AZ proteins have to interact coordinately to accomplish normal and dynamic synaptic functions. Altogether, AZ proteins, the cytoskeleton, and adhesion and signaling molecules maintain the integrity of the presynapse.

**Figure 3:**
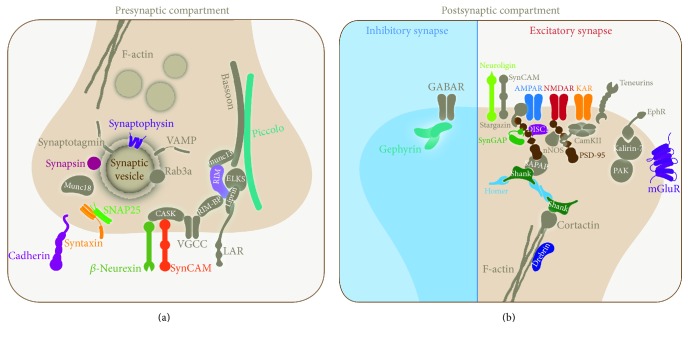
Schematic representation of synaptic proteins associated with synaptopathies. (a) Presynaptic and (b) postsynaptic proteins involved in human synaptopathies described in this review are color highlighted. Mutations in a gene or gene combination for a synaptic protein may lead to neurodevelopment, neuropsychiatric, and neurodegenerative diseases.

A group of proteins that are directly involved in the exocytosis of SVs is the SNARE (SNAP Soluble NSF Attachment Protein REceptor) complex formed by synaptobrevin, syntaxin, and synaptosomal-associated protein 25 (SNAP25), which mediates SV fusion with the AZ plasma membrane [18]. Syntaxin and SNAP25 are plasma membranes proteins and synaptobrevin is a SV protein. Other SV proteins, synaptotagmin, synaptophysin, and synapsin, participate in different steps of exocytosis. A second group of AZ proteins that form the cytomatrix at the active zone (CAZ) has been highly evolutionarily conserved: Rab3 interacting molecules (RIM Munc13, ELKS, RIM-binding protein (RIM-BP), and liprin-*α*) suggesting a primordial role at presynapses [2]. In fact, this group of proteins functions in SV priming, docking, calcium channel localization and clustering, and scaffolding. Other CAZ proteins are Piccolo and Bassoon, two large, highly homologous vertebrate AZ proteins with roles in scaffolding and synaptic integrity [19]. In addition, Bassoon plays a role in SV endocytosis at CNS synapses [20] and in calcium channel clustering at ribbon synapses in the retina and cochlea [21]. Interestingly, Piccolo participates in the dynamic assembly of F-actin to regulate the migration of SVs to the AZ [22]. Presynapses also contain adhesion molecules, which, in addition to mediating cell-cell contacts, also deliver intracellular signaling through trans-synaptic communication. Here, we review some presynaptic proteins that have been associated with synaptopathies in genome-wide association (GWA) studies and family linkage studies (Figure 3(a) and Table 1).

### 2.1. Synaptic Vesicle Proteins

#### 2.1.1. Synapsin

Synapsins are phosphoproteins that are associated with the membrane of SVs and play a role in tethering SVs to the cytoskeleton away from the AZ. The phosphorylation of synapsin during an action potential induces the release of SVs from the reserve pool, allowing their movement toward the presynaptic AZ to release neurotransmitter. Therefore, synapsin will regulate the number of vesicles accessible for exocytosis. In vertebrates, three synapsin genes have been described (*SynI/SynII/SynIII*) [69] that are alternatively spliced to render the 2–6 protein isoforms [70, 71]. Synapsins have been implicated in several psychiatric disorders, such as BPD and SCZ [23], and specific mutations and polymorphisms in* Syn* genes cause familial epilepsy [24, 31]. Accordingly, a causal role has been attributed to* SynI* and* SynII* in the pathogenesis of ASD and epilepsy [24, 25]. Like nonsense mutations, Q555X in the* SynI* gene was found in a family presenting both ASD and epilepsy [25]. The expression levels of synapsin also seem to correlate with psychiatry disorders because a decrease in synapsin-2a and synapsin-3a has been observed in the hippocampal tissue of patients with SCZ and BPD [23, 26] and decreased protein levels of synapsin-2a were observed in the olfactory bulbs from people with SCZ [27]. In another study, a decrease in* SynII* gene expression in postmortem brain tissue of BPD patients might be explained by the presence of hypomethylated I CpG islands found in this gene [32]. The findings in humans are, in part, reproducible in animal models because a* Syn II* knock-out (KO) animal model results in a schizophrenic-like phenotype [28–30]. The third member of the family, synapsin 3, has also been implicated in SCZ because a decrease in its expression was observed in the prefrontal cortex of individuals with SCZ [33].

#### 2.1.2. Synaptophysin

Synaptophysin is a SV glycoprotein and the most widely used synaptic marker. Interestingly, KO mice for synaptophysin are normal, but electrophysiological experiments indicate that this protein is necessary for efficient endocytosis of SV in hippocampal neurons [72]. Synaptopathies involving synaptophysin are less evident because studies investigating these diseases are discrepant. In a recent study of the CA1 region of the hippocampus derived from a postmortem individual with SCZ, synaptophysin levels were decreased together with PSD95 and Homer [34]. These molecular defects at synapses in the CA1 region of the hippocampus might explain, in part, the cognitive defects in SCZ. Synaptophysin also might participate in the pathology of BPD. Scarr et al. studied the expression of several proteins involved in SV exocytosis in Brodmann area 9 of the brain cortex of a subject with BPD [35], an area of the brain with lower levels of energy consumption in bipolar patients [73]. In that study, increases in SNAP25 and synaptophysin were observed, suggesting a role for these proteins in this disease [35].

### 2.2. Cytomatrix of Active Zone Proteins

#### 2.2.1. RIMs

RIMs were first identified as Rab3-interacting molecules [74], among which there are four isoforms (RIM1-4) encoded by different genes in vertebrates. RIM proteins are represented by two long isoforms, RIM1*α* and RIM2*α*, which contain a zinc-finger domain, a PDZ, and two C-terminal C2A and C2B domains [75]. Short isoforms for RIM include RIM2*γ*, RIM3*γ*, and RIM4*γ* formed by the C2B domain. RIM1*α* participates in SV docking and priming and the recruitment of voltage-dependent Ca^2+^ channels (VDCCs) into the AZ of presynapses. RIM1*α* also plays a role in presynaptic long-term potentiation (LTP) in the hippocampus and cerebellum [76]. The RIM short isoform, RIM3, is postulated to have a role in the regulation of neurotransmitter release by modulating presynaptic Ca^2+^ influx [77]. Deletion of the* Rims1* and* Rims2* genes, which produce the five isoforms, RIM1*α*, RIM1*β*, RIM2*α*, RIM2*β*, and RIM2*γ*, in mice severely impairs the Ca^2+^ responsiveness of neurotransmitter release via a mechanism that affects Ca^2+^ channel tethering to the AZ [78]. A single gene deletion produced a mild effect, suggesting a redundant and compensatory role for these two genes [78].

Although an association with human SCZ has not been attributed to RIM1*α*, KO mice for this protein exhibit a phenotype similar to human SCZ [36]. Interestingly, a genetic microdeletion and a genome-wide expression profiling study revealed an association of the RIM3 isoform with ASD in children with this disease [38, 39]. Increased expression levels of RIM2 and RIM3 were observed in the amygdala in SCZ [37]. Recently, a role in axonal and dendritic arborization was assigned to RIM3 and RIM4 [79]; thus, the participation of these proteins in psychiatry disorders is not rare because autism and SCZ are diseases associated with aberrant dendritic growth and alterations of dendritic spine numbers [80].

#### 2.2.2. Piccolo

Piccolo is a multidomain CAZ protein and the largest protein in the presynapse. Piccolo is a nontransmembrane protein that is transported during development into newly forming synapses in a dense core vesicle of Golgi origin [81, 82]. Piccolo interacts with several actin binding proteins, including Abp1, GIT-1, and PRA1 [19], and it is thought that through these interactions Piccolo modulates F-actin dynamics at presynapses [83]. In the AZ, Piccolo forms a macromolecular complex with other AZ proteins, including Bassoon, ELKS, RIM, and Munc13 [19]. Recent studies have shown that Piccolo and Bassoon regulate the stability of AZ proteins at presynapses [84], and hence a defect in these proteins might compromise the structure and function of synapses in synaptopathies.

In population studies, Piccolo has demonstrated an association with some psychiatric disorders. Weidenhofer et al. reported an increase in the gene expression of* PCLO*,* Rims2*, and* Rims3* in the amygdala in SCZ [37], although some variability in Piccolo expression was observed, suggesting that this protein might not be affected in some cases of schizophrenia. A GWA study conducted in the Netherlands [40] and corroborated by others [41, 42] found an association of the* PCLO* gene with MDD due to a single nucleotide polymorphism (SNP), SNP rs2522833, in which a serine is replaced with alanine in the C2A domain, a region known to bind phosphatidylinositol or synaptotagmin-1. Following this finding, Furukawa-Hibi et al. [85] produced a transgenic mouse overexpressing the Piccolo C2A domain. Interestingly, the animals presented depression-like behavior, supporting the hypothesis that disruption of the interactions of the C2A domain for Piccolo with other presynaptic proteins can cause depressive behavior. The participation of Piccolo in mood disorders is also supported by another study examining the genetic variation of individuals with BPD, in which two SNPs were found in an intron of the* PCLO* gene accompanied by increased expression of the protein [43]. Therefore, mutated or unbalanced expression of Piccolo might trigger psychiatry disorders.

### 2.3. SNARE Proteins

#### 2.3.1. SNAP25

SNAP25 is a t-SNARE protein and a key component of the SNARE protein complex, the machinery involved in the fusion of SVs. SNAP25 assembles with syntaxin and synaptobrevin to mediate vesicle docking and Ca^2+^-triggered fusion.

SNAP25 has been involved in several human neuropsychiatric disorders. Abnormal levels of the protein have been found in the postmortem brain of bipolar patients [35, 44], and a SNAP25 variant was found in the prefrontal cortex of patients with early-onset BPD [45]. In a linkage study, two polymorphisms located in the 3′-untranslated region of the human* SNAP* gene were associated with ADHD [48]. Additional evidence for a role of SNAP25 in ADHD was provided by a recent meta-analysis [49]. In SCZ, the structure of the hippocampus is abnormal, and a decrease in the amount of SNAP25 has been observed in the hippocampus and areas of the cortex of patients with this disease [44, 46, 47]. The same has been observed in the hippocampus of animal models of ADHD [50]. It has been postulated that abnormal levels of SNAP25 expression affect neurotransmitter release mechanisms and short-term plasticity, which are characteristics of SCZ and ADHD.

The role of SNAP25 in neuropsychiatry disorders might not only be explained by its novel function in exocytosis because recent articles have assigned postsynaptic functions to this SNARE protein. Accordingly, SNPA25 was assigned a role in NMDAR and kainate-type receptor trafficking [86, 87]; however, no studies have demonstrated its presence at dendritic spines. The findings of Tomasoni et al. support a role for SNAP25 in postsynaptic function by demonstrating structural modifications at the PSD and immature dendritic spines upon SNAP-25 diminution [88]. Interestingly, as previously mentioned, SCZ is associated with defects in spine morphology and dynamics, and some SNAP25 mutants are related to this disease.

### 2.4. Adhesion Molecules

#### 2.4.1. SynCAM1

Synaptic adhesion molecules (SynCAMs) are molecules that actively participate in synapse formation and plasticity [89, 90] and axonal pathfinding [51]. They are a subgroup of the immunoglobulin superfamily of cell adhesion molecules that are localized both pre- and postsynaptically and mediate homophilic cell-cell interactions in a Ca^2+^-independent manner. Two missense mutations in the* SynCAM1* gene were found in DNA samples of individuals with ASD [52]. These two mutations were found in a domain necessary for trans-active interactions. In addition, the mutant proteins were more susceptible to protease cleavage and presented abnormal intracellular trafficking [52]. The role of SynCAM1 in this pathology is strengthened by investigations of SynCAM1 KO mice showing impairment in social behavior similar to individuals with ASD [53, 54]. The role of SynCAM in axon guidance during development and its contribution to neural circuit formation might explain its participation in the pathology of ASD [51].

#### 2.4.2. Cadherin

Cadherin is a Ca^2+^-dependent homophilic cell adhesion molecule with a role in neuronal target selection, synapse formation, and plasticity in the vertebrate CNS. It comprises a superfamily of approximately 100 members expressed in brain. In the CNS, cadherin expression follows a spatiotemporal pattern, suggesting an important role in specific circuit development [91]. Classic cadherins are found in pre- and postsynaptic compartments, at both nascent and mature synapses [92, 93]. The structures of classic cadherins include 5 extracellular EC repeats and an intracellular binding domain, and they are encoded by 20 genes. At presynapses, the intracellular domain of classic cadherin interacts with *β*-catenin, which serves as a linker to F-actin [94].

Several neuropsychiatric diseases have been associated with cadherins, such as SCZ, ASD, BPD, and alcoholism [55]. Here, we provide a few examples. Genetic microdeletion of cadherin-8 and duplication of cadherin-13 were found in individuals with ASD and learning disability [57, 58]. A recent study aimed at identifying common genetic risk factors for ASD studied 780 families with children with ASD. They found six single SNPs in the cadherin-9 and cadherin-10 genes, suggesting an association of the adhesion molecules with autism [59]. In addition, the deletion of cadherin-12 and cadherin-18 genes was correlated with SCZ [56]. These observations of defects in cadherin genes in patients with autism have also been observed in population studies in which the presence of these SNPs is correlated with individuals with problems in oral communication but not spatial memory [60, 61]. These findings suggest that cadherins may be involved in the regulation of specific circuits related to verbal working memory.

#### 2.4.3. Neurexins

NRXNs are presynaptic adhesion molecules that interact transsynaptically with postsynaptic neuroligins (NLs), an interaction that is known to be important for synaptogenesis and synapse maintenance [95]. NRXNs are encoded by three genes (*NRXN1-3*), each of which has two promoters that generate long (*α*) and short (*β*) protein isoforms with identical intracellular but different extracellular domains. The distribution of NRXNs in the CNS has been studied by in situ hybridization. The data suggest their differential expression in the embryonic nervous system but partial overlap in the mature central nervous system [96]. However, protein expression experiments that also consider splice variants are needed to decipher the precise distribution of these proteins [97, 98].

Studies conducted* in vitro* have shown that both NRXNs and NLs can induce postsynaptic or presynaptic specialization clustering, respectively [99, 100]; however, these findings have not been completely observed in loss of function animal models. Consequently, more work is necessary to understand the synaptogenic role of NRXN-NL. NRXNs interact through their intracellular domain with CASK and Mints, two proteins that are known to interact with the *β*-subunit of N-type Ca^2+^ channels and with P/Q-type Ca^2+^ channels in the case of Mints [101]. This interaction might link NRXNs to the SV release machinery [102].

The first report linking NRXN to a neurodevelopmental disorder was performed in a boy with ASD in whom the promoter and exons 1–5 were deleted from the* NRXN1* gene encoding NRXN-1*α* [103]. Other investigators have observed similar deletions in the NRXN 1*α* gene in ASD [62–65]. All these deletions were heterozygous for NRXN-1*α*. Deletions in* NRXN1* have also been identified in SCZ patients and involve the promoter and exon 1 of NRXN 1-*α* [66–68]. No postmortem studies have been performed in ASD and schizophrenic individuals carrying a deletion in the* NRXN*-*1α* gene, and thus these findings cannot be correlated to the expression levels of the protein. A homozygous KO mouse has been developed for the* NRXN1* gene [104]. Although this animal model does not represent heterozygous ASD and schizophrenic human cases, these animals showed mild behavioral deficits similar to those observed in ASD and SCZ individuals [105]. Furthermore, the* NRXN-1α* KO mice showed impairments in social interaction and communication analogous to those observed in ASD [104, 106]. Taken together, we can conclude that NRXN plays a role at the synapse and that further knowledge of the functional interactome of NRXN might improve our understanding of its specific functions and roles in synaptopathies.

## 3. Postsynaptic Proteins

The PSD is a dynamic lattice-like array composed of interacting proteins lining the postsynaptic membrane that organize and stabilize synaptic receptors, ion channels, structural proteins, and signaling molecules required for normal synaptic transmission and synaptic function [107, 108]. However, its composition and morphology are dynamically changing as a function of neuronal activity, and thus the PSD plays a fundamental role in regulating the strength and plasticity of excitatory synaptic neurotransmission [109, 110]. Therefore, maintenance of the architecture and composition of the PSD is considerably important for proper synaptic connections that allow the preservation of cognition, memory, and functional circuitry. In most neurological diseases, one or more of these processes are disrupted and impaired. Consequently, an understanding of the PDS molecular network and signaling pathways underlying normal synapse function is crucial to comprehend the pathological mechanisms responsible for different synaptopathies. Here, we describe some postsynaptic proteins that are involved in synaptopathies (Figure 3(b) and Table 2).

### 3.1. Adhesion Molecules

#### 3.1.1. Neuroligins

NLs are postsynaptic cell adhesion proteins that participate in associations with presynaptic NRXNs in synaptogenesis through the recruitment to synaptic sites of receptors, channels, and signaling molecules. NLs constitute a multigene family of brain-specific membrane proteins composed of different isoforms in humans, including NL1, NL2, NL3, NL4, and NL4Y (occasionally referred to as NL5) [98, 216]. Although it has been suggested that NLs may develop similar functions related to mediating recognition processes between neurons, sequence comparisons have shown that NL1, NL3, and NL4 are more similar to one another than to NL2 [12]. Several studies have reported that NL1 is particularly localized to excitatory synapses and interacts with postsynaptic density protein-95 (PSD-95), which is highly enriched at the PSD [217]. NL3 and NL4 are similarly expressed at excitatory synapses [95], but they have also been found in inhibitory ones: NL3 at *γ*-aminobutyric acid- (GABA-) ergic [218] and NL4 at glycinergic synapses [219]. In contrast, NL2 is located exclusively at inhibitory synapses and clusters with GABA_A_ and glycine receptors [220, 221]. Although NL2 is present specifically at inhibitory synapses, NL2 has revealed excitatory properties in a spinal nerve ligation model of neuropathic pain through a functional shift from inhibition to excitation [222].

NLs are well-accepted molecules that participate in the pathogenic mechanism of diverse neurological diseases and exert a strong genetic influence on developmental disorders. It has been reported that an aberrant form of NL at the postsynaptic membrane, an anomalous association with NRXN, or both anomalies trigger an abnormal excitatory and inhibitory balance and the underlying development of cognitive disorders.

A proper NL1 level, especially in the hippocampus, is crucial for memory formation. Moreover, impairment of the NL1 level might induce the development of autism-related symptoms [111, 112] and also participate in the cognitive disability observed in AD [223]. Particularly during the early phases of AD, it has been reported that A*β* oligomers preferentially bind to postsynaptic regions where they might interact with* N*-methyl-D-aspartate (NMDA) receptors (NMDARs) and NL1 [224, 225]. Moreover, NL1 has been reported to act as a nucleating factor in the stabilization of A*β* accumulation* in vitro* by inducing the formation of A*β* oligomers [226]. These data suggest that NL1 can promote the targeting of A*β* oligomers to the postsynaptic sites of excitatory synapses and thereby promotes synaptic toxicity in AD. A mutation in the* NL1* gene was found in AD patients, generating a premature stop codon in the extracellular domain of* NL1* (p.Thr271fs) that blocks the function of NL1 and thus its ability to form glutamatergic synapses [113]. Interestingly, in a neuroinflammation rodent model induced by hippocampal injections of A*β*_1–40_, there was a decrease in NL1 expression with subsequent impairment of synaptic function and memory [227, 228]. All of these studies indicate that altered NL1 function could underlie the molecular mechanisms associated with the memory loss and the cognitive impairment observed in AD patients.

In addition to its involvement in AD pathology, NL1 is known to participate in molecular mechanisms related to other neurological diseases [229]. The role of NL1 in Fragile X syndrome (FXS), the most common form of inherited mental retardation, is supported by a study conducted in a mouse model of the disease, in which the overexpression of NL1 improved social behavior without any observed effect on learning and memory [114].

Several studies have linked NL2 with symptoms related to neurological diseases, such as anxiety and SCZ, or alterations in normal behavior. In a genetic study of 584 SCZ patients, several mutations were found in the* NL2* gene, two of which were related to abnormal GABAergic synapse formation, suggesting a role in the onset of SCZ [115]. Studies in* NL2*-deficient mice have reported that* NL2* deletion disrupts the inhibitory synapse function in hippocampal sections without affecting their numbers and also triggers a pronounced anxiety phenotype in those mice [230]. In addition,* NL2* deletion also affects inhibitory synapses in projection neurons of the basal amygdala, leading to their excessive activation under anxiogenic conditions [231]. Overexpression of* NL2* caused different social and emotional behaviors in rats such as reduced aggression [232], whereas transgenic mice exhibited a stereotypical jumping behavior, anxiety, and impaired social interactions, probably as a consequence of a significant increase in the density of inhibitory synapses and the subsequent morphological change in the excitatory synapse [233]. To study the role of NL2 in the pathogenesis of ASD, Wöhr et al. investigated the presence of several behavioral phenotypes observed in ASD patients in* NL2* null and heterozygote mice [116]. These mice presented some of the behavioral characteristics of ASD patients, suggesting that NL2 plays a partial role in the etiology of the disease.

For the* NL3* and* NL4* genes, point mutations, truncations, and sequence deletions in their coding regions are associated with both ASD and mental retardation [117–119]. In some patients with ASD, the Arg^451^ residue is substituted by Cys^451^ (R451C) in NL3. Interestingly, NL3 (R451C) mutant mice exhibit a deficit in social behaviors and learning abilities as a consequence of inhibited synaptic transmission in addition to a significant increase in *α*-amino-3-hydroxy-5-methyl-4-isoxazolepropionic acid (AMPA) receptor- (AMPAR-) mediated excitatory synaptic transmission, enhanced NMDAR-NR2B regulation, and increased LTP. Moreover, this mutation alters dendritic branching with consequent alterations of the structure of the synapse [120]. Regarding the effect of NL3 (R451C) in GABAergic postnatal signaling in the hippocampus, it has been reported that this knock-in mutation produces enhanced GABAergic but not glutamatergic transmission, suggesting that NL3 regulates the excitatory/inhibitory balance during the development of neuronal circuits [121]. Conversely, another study showed that the NL3 (R704C) mutation triggered impaired synapse function, specifically inducing a decrease in AMPAR-mediated synaptic transmission in hippocampal slices without changing NMDA or GABAR-mediated synaptic transmission [105], thus suggesting that NL3 plays a fundamental role in synaptic transmission in excitatory synapses.

Several deletions of X-chromosomal DNA in the* NL4* locus have been found in a wide spectrum of neuropsychiatric conditions and in autistic and nonautistic mentally retarded patients [122, 123]. Moreover,* in vitro* studies have also revealed that* NL4X* deletion results in neurodevelopmental defects during the formation of neurons and their connections as well as in decreased gene expression of NL1 and NL3 [124]. The amino acid R87 is conserved in all NL isoforms, but a single amino acid substitution in NL4 (R87W) that affects the activity of NL4 in synapse formation and abolishes the functional effect of NL4 on synapse strength has been discovered in ASD patients [125]. In addition, a single mutation has been identified in NL4X (R704C) that consists of a single amino acid substitution in a conserved arginine residue. As previously mentioned, this mutation has been studied in a homologous recombination mouse NL3 model, in which the analogous arginine is conserved [105]. Furthermore, the R704C mutation, which is very close to the T707 residue that is phosphorylated in humans by protein kinase C (PKC) [126], inhibits the phosphorylation of T707 through an unknown mechanism. Additionally, the phosphomimetic mutation at T707 has demonstrated enhanced synaptogenesis, possibly via an unknown mechanism that includes glutamatergic receptors or presynaptic terminal recruitment [216].

### 3.2. Glutamate Receptors

Glutamate is considered the major excitatory neurotransmitter in the human brain, and the pathophysiology of several mental disorders is known to depend on glutamatergic system activity. Glutamate receptors comprise the ionotropic (iGluRs) and metabotropic glutamate receptors (mGluRs). iGluRs include NMDA, AMPA, and kainate receptors based on structural, pharmacological, and physiological properties.

#### 3.2.1. *N*-Methyl-D-aspartate Receptors, NMDARs

NMDARs are formed by three subunits called GluN1-3 and different splice variants [234]. NMDARs consist of tetrameric structures with a large number of receptor subtypes that determine their pharmacological and functional properties. These receptors are crucial for neuronal communication and are recognized to have a key role in neural plasticity.

Several lines of evidence indicate that NMDARs are involved in different ASDs. De novo mutations in the GluN2B (*GRIN2B*) and GluN2A (*GRIN2A*) genes have been identified in different cases of ASD and SCZ, respectively, as well as truncation mutations in* GRIN1*,* GRIN2B,* and* GRIN2A* in ASD and SCZ patients [127]. Animal models have allowed the development of suitable ASD-related phenotypes, such as the parvalbumin-selective* NR1* KO, which results in reduced sociability and impaired ultrasonic vocalizations [128]. Moreover, the use of NMDARs antagonists, such as ketamine [235] or D-cycloserine [236], has contributed to testing therapeutic drugs in patients or animal models of ASD [129]. Typical behavioral manifestations and cognitive impairment of SCZ have been associated with dysfunctional NMDAR trafficking and regulation [130], which is indeed regulated by different genes. For example, it has been reported that the stimulation of neuregulin-1, a growth factor associated with SZC in humans [237], triggers a rapid internalization of NMDARs, suppressing their activation in the postmortem prefrontal cortex of SCZ patients [131].

In AD patients, the glutamatergic system, especially NMDAR-mediated transmission, appears to be strongly affected because NMDARs are activated by the accumulation of A*β* oligomers during the initial phases of the disease [132, 133]. In particular, it has been reported that A*β* oligomeric species activate the GluN2B subunit of NMDARs, which in turn produces an increase in intracellular Ca^2+^ levels and subsequent excitotoxicity [134]. Conversely, it has been reported that oligomeric A*β* causes a selective loss of synaptic GluN2B responses together with a subunit composition from GluN2B to GluN2A [135], potentially as an attempt to reduce A*β*-induced injury because GluN2A subunits are implicated in protective signaling pathways [238]. Several NMDAR antagonists, such as 1-benzyl-1,2,3,4-tetrahydro-*β*-carboline [239] or MK-801 [240], have been generated and used as potential therapeutic drugs to prevent synaptic dysfunction in AD models. However, it is noteworthy that, considering the involvement of NMDARs in synaptic function, complete inhibition of their activity triggers important secondary effects such as severe memory impairment. Interestingly, memantine, a low-affinity NMDAR antagonist that is also employed for the treatment of dementia and depression, does not accumulate in the channel, allowing normal synaptic transmission [241]. However, it is well-accepted that the hyperphosphorylated tau protein contributes to the AD-associated neurodegeneration and is also required for the A*β*-mediated neurotoxicity [242]. Some studies utilizing transgenic mice have confirmed that both A*β* and tau are involved in the neuropathology of AD. For example, A*β* has been shown to function via NR2A to trigger dendritic spine loss, whereas tau acts through the NR2B subunit to promote neurodegeneration [136].

Similarly, several HD transgenic mouse models have indicated that NMDARs, as well as the GluN2B subunit, are involved in the pathology of HD. The motor learning deficits manifested by YAC128 mice expressing the mutated Htt (mHtt) were attenuated by chronic extrasynaptic NMDAR blockade with memantine [137, 138]. In addition, the mechanism of action of an antihistamine compound proposed for the treatment of different neurological diseases including HD, Dimebon, has been reported to occur through the inhibition of NMDAR activity [243].

#### 3.2.2. Kainate Receptors, KARs

Kainate receptors (KARs), which are highly expressed in the cortex and hippocampus, are targeted to synapses, where they play specific roles in the maturation of neural circuits during development [244]. KARs are tetrameric receptors that form homomeric or heteromeric receptors via the combination of five subunits: GluR5 (GRIK1), GluR6 (GRIK2), GluR7 (GRIK3), KA1 (GRIK4), and KA2 (GRIK5).

Some abnormalities in genes encoding the glutamate receptor subunits of the kainate type, such as* GRIK2* and* GRIK4*, have been reported to be involved in BPD, SCZ, ASD, and mental retardation diseases [139, 142, 143]. In particular, chromosome 6q21 has been identified as an important region for autism, and a SNP was found in the glutamate receptor 6 (*GluR6* or* GRIK2*) gene related to ASD [140]. Additionally, overexpression of* GRIK4*, a gene encoding KA1, induced an altered synaptic transmission in mice that also manifested ASD-associated symptoms such as enhanced anxiety, depressive states, and impaired social interactions [141]. Despite this association of KARs with the pathology of neurological diseases, additional studies are necessary to fully understand their roles in synapse function.

#### 3.2.3. *α*-Amino-3-hydroxy-5-methyl-4-isoxazolepropionic Acid Receptors, AMPARs

AMPARs are glutamate receptors that mediate fast synaptic transmission in the CNS. They are formed by the heterotetrameric combination of four subunits: GluR1-4, which determine the functional properties of AMPARs.

A deletion mutation in the* AMPA 2* gene encoding the glutamate receptor GluR2 subunit has been found in patients with ASD [144]. Furthermore, AMPARs have been associated with SCZ through dysbindin, a widely suspected susceptibility protein in SCZ. Accordingly, enhanced AMPAR-mediated transmission has been observed in cultured hippocampal neurons from dysbindin-deficient mice [145]. However, several unsuccessful genetic studies in humans have been developed to associate different SNPs in the AMPAR subunits, GluR1, GluR2, and GluR4 (encoded by the* GRIA1*,* GRIA2,* and* GRIA4* genes, resp.), with SCZ [146], BPD [148], and major depressive disorder [149]. Similarly, although altered trafficking of AMPAR has been associated with the pathology of SCZ, no changes were observed in the expression of the AMPAR subunits GluR1-4 in the endoplasmic reticulum of SCZ patients [245].

However, a role for AMPAR in the pathology of SCZ cannot be discarded because olanzapine, an atypical antipsychotic drug, has a therapeutic effect on memory dysfunction and cognitive impairment manifested in SCZ patients through the modulation of synaptic plasticity caused by the upregulation of GluR1 Ser845 phosphorylation [147]. In addition, 7,8-dihydroxyflavone, a tropomyosin receptor kinase B (TrkB) agonist, is considered a potential pharmacotherapeutic strategy for FXS. Briefly, FXS is a common inherited cause of mental retardation, human cognitive dysfunction, and autism resulting from the transcriptional silencing of the FMR1 gene that encodes FMRP. The 7,8-dihydroxyflavone induces an increase in GluR1 subunit expression that results in improved spatial and fear memory and a decrease in morphological abnormalities in the spines of* Fmr1* KO mice [150]. Furthermore, another link between AMPARs and FXS has been reported, in which FXS-associated synaptic proteins also regulate the AMPAR subunit GluR1, supporting an important role in neuronal development and maturation [151].

Therefore, several lines of evidence indicate that the proper function of AMPARs, the major mediators of excitatory transmission in the CNS, is highly important for synaptic plasticity and cognitive functions, as evidenced by the association with several and different neurological disorders including ASDs, SCZ, AD, FXS, and HD [152].

#### 3.2.4. Metabotropic Receptors, mGluRs

mGluRs are involved in the regulation of neuronal excitability, learning, and memory and are classified in three groups as follows: mGluR1 and mGluR5 belong to the group 1 family; mGluR2, mGluR3, and mGluR4 form the group 2 family; and mGluR6, mGluR7, and mGluR8 are included in the group 3 family. These receptors are found synaptically and extrasynaptically. Group I members, which comprise mainly postsynaptic receptors that activate neuronal depolarization and excitability, are coupled to Gq/G11 and activated phospholipase Cb to generate inositol 1,4,5-triphosphate (IP3) and diacylglycerol with the consequent mobilization of calcium and activation of protein kinase C (PKC). In contrast, groups 2 and 3 members are mostly presynaptic receptors that are localized in positions where they inhibit synaptic vesicle release through Gi/o proteins.

Some evidence indicates that mGluRs are involved in both nonsyndromic [153] and syndromic cases of autism [154]. In a valproate-induced rat model of autism, a significant reduction of mGluR2/3 protein and mRNA levels has been observed [155].* N*-acetylcysteine, a drug that stimulates the uptake of cysteine in exchange for glutamate, which is transported to the extracellular milieu through the antiporter Xc-, reverted the social interaction and anxiety behaviors of autistic rats as a result of presynaptic mGluR2/3 [155]. In addition, mGluRs are also associated with FXS. The reduced mGluR5 expression has been observed to impede the development of abnormalities in* Fmr1* KO mice [157]. Furthermore, a recent study confirmed that mGluR5 dysfunction is associated with neurological disorders such as obsessive-compulsive disorder and autism [156]. Conversely, enhanced mGluR5 function is associated with FXS pathology. In* Fmr1* KO mice, mGluR5 was shown to have a weaker association with its scaffolding protein Homer, which targets mGlu5 to synapses, and thus disrupted mGluR5-Homer scaffolds triggered dysfunctional mGluR5 and FXS phenotypes [158]. A recent study reported that, in transgenic mice expressing mutant mGluR5 unable to bind Homer, the most representative biochemical, neurophysiological, and behavioral alterations of the illness were observed in FXS mice [159].

### 3.3. Scaffolding Proteins

#### 3.3.1. PSD-95

The postsynaptic density protein 95 (PSD-95; also known as DLG4 and SAP90) is the most abundant protein in excitatory chemical synapses and the main scaffolding protein at the PSD. In fact, PSD-95 contributes to synaptic stabilization, strength, and transmission, and its proper regulation is known to be essential for accurate synaptic development and plasticity. PSD-95 belongs to the membrane-associated guanylate kinase (MAGUK) protein family. All of these proteins possess three independent PDZ (PSD-95, Dlg1, and zonula occludens-1 proteins (zo-1)) domains through which they interact with glutamate receptors, cell adhesion molecules, and cytoskeletal elements. The PDZ domain of PSD-95 binds to different postsynaptic proteins; for example, PDZ2 interacts with NR2 and NR1 of NMDARs [246] and stargazin interacts with PDZ1 and PDZ2 of PSD-95 to regulate AMPAR synaptic numbers [247]. Other proteins, such as Ras GTPase-activating protein (SynGAP), appear to interact with the three PDZ domains of PSD-95 [248]. In addition to making PSD-95 a target of multiple synaptic interactions, PDZ domains confer to this protein a high susceptibility to modification by posttranslational modifications that affect its postsynaptic localization within the PSD in dendritic spines. However, alterations of some of the signaling pathways that control these modifications may contribute to the development of neurological diseases such as AD, SCZ, HD, and FXS [249].

Synaptic alterations in AD are often correlated with cognitive changes. Regarding the association between the alterations in PSD-95 and AD, it is known that during brain aging, A*β* oligomers may bind directly to NMDARs, which in turn interact with PSD-95 [224, 225]. Moreover, an altered distribution pattern of NMDARs and PSD-95 has been observed in human AD postmortem brains [160] and a direct relationship between A*β* oligomers and PSD-95. In fact, it has been reported that A*β* oligomers colocalize with PSD-95 at excitatory synapses in AD brain tissues as well as in cultured rat hippocampal neurons exposed to A*β* oligomers [250, 251]. A deleterious effect of A*β* oligomers is known to increase with time. The latter could be explained by the time-dependent decrease in the levels of synaptic PSD-95 in excitatory synapses as the pathology advances in murine models of AD, suggesting that this PSD-95 reduction is a sign of the postsynaptic degeneration underlying long-term functional deficits [161, 162]. The specific mechanism by which PSD-95 expression is reduced in patients with AD may result from the ubiquitin-proteasomal degradation of PSD-95. It has been reported that in cells transfected with a PSD-95 mutant lacking the PEST sequence, which is essential for its ubiquitination, A*β* treatment fails to decrease the expression of PSD-95 [252]. Therefore, the regulation of PSD-95 would be a crucial step in the pathological progression mediated by A*β* oligomers. Accordingly, it has been reported that Wnt-5a, a synaptogenic ligand, decreases the synaptic disruption induced by A*β* oligomers, revealing its neuroprotective role by blocking the reduction of synaptic PSD-95 in hippocampal neurons exposed to A*β* oligomers [253]. Therefore, in AD neuropathology, the ability of PSD-95 to interact with other synaptic elements is impaired with the consequent disruption of the organization and stabilization of the PSD, resulting in a loss of NMDARs and SynGAP [254]. Therefore, molecules involved in different signaling pathways that regulate PSD-95 may have therapeutic potential for decreasing A*β*-induced synaptic loss and cognitive impairment in AD.

The role of PSD-95 in the etiology of ASD is less clear because no rare genetic mutations in PSD-95 have been associated with ASDs to date; however,* PSD-95* deletion mice exhibit behavioral and molecular abnormalities that are related to ASD symptoms, such as increased repetitive behaviors, altered social behaviors, impaired motor coordination, and anxiety [163].

Reduction of the hippocampal size is one of the characteristics of SCZ patients, and it has been reported that the CA1 region of the hippocampus plays an important role in the pathophysiology of SCZ. In postmortem brains of SCZ patients, the expression of PSD-95 is reduced together with its known interacting proteins Homer1 and mGluR1 [34]. Therefore, the molecular abnormalities in PSD-95 and its molecular interactome may contribute to the cognitive dysfunction displayed in SCZ patients [34, 164, 165]. Similarly, altered levels of PSD-95 have been observed in mouse models of HD [166, 167]. In these patients, the Htt is structurally altered and associated with a loss of function. Using a knock-in mouse model of HD, characteristic motor and cognitive deficits of the illness were observed in mutant mice, as well as altered levels of PSD-95 and other proteins associated with synaptic function [168]. All of these studies indicate that mHtt contributes negatively to synaptic plasticity and may be one of the mechanisms underlying the cognitive deficits in HD.

It is known that PSD-95 possesses a binding site for FMRP, the FXS-related protein, and that its translation depends on the absence of this protein in FXS patients [169]; moreover, FMRP is also required for the stability of PSD-95 [255, 256] and glutamate receptor mRNA in the PSD [257]. Furthermore, FMRP is essential for correct excitatory synapse elimination through the proteasomal degradation of PSD-95, and thus the defective degradation of PSD-95 might explain the excessive number of dendritic spines observed in patients with FXS [170]. Several studies conducted in FXS mouse models have reproduced the phenotype observed in FXS patients, including deficits in cognitive flexibility, attention, and inhibitory control. In addition, a strong relationship has been observed between the decreased levels of proteins involved in synaptic function, such as PSD-95, and cognitive impairment [171–173]. A recent study has reported that FMRP colocalizes with PSD-95 [258]. Therefore, PSD-95 is clearly associated with the pathobiology of FXS.

In summary, any alterations of the synaptic levels of PSD-95, a key molecule in PSD organization and function, may affect interactions with its partners and contribute to the development of several CNS diseases.

#### 3.3.2. Shank Family Proteins

The members of the Shank/ProSAP family, Shank1, Shank2, and Shank3, are multidomain scaffold proteins located at the PSD of glutamatergic synapses that interact with a large variety of membrane and cytoplasmic proteins. Shank proteins are expressed in areas of the brain that are essential for cognition and learning and trigger a crucial role in spine formation and maturation [259]. Shanks have differential patterns of expression in the CNS: Shank2 is the first isoform expressed in the brain, followed by Shank3 and then Shank1 [260]. Shank proteins interact with a large number of postsynaptic proteins to regulate PSD function, including ionotropic-glutamate receptors, PSD-95, Homer, and components of the actin cytoskeleton [261]. Furthermore, the trans-synaptic functions of Shanks are believed to be modulated by their interaction with the NL-NRXN complex [262].

Human genetic studies have strongly linked Shank genes, including* Shank1*,* Shank2*, and* Shank3*, with Phelan-McDermid syndrome (PMS), ASD, AD, and SCZ associated with ID [250, 260, 263]. A rare inherited deletion encompassing the* Shank1* gene has been associated with autism in males, whereas only anxiety and shyness behaviors were observed in females, suggesting that* Shank1* deletion could have sex-dependent effects [174]. Several genetic mouse models have been used to study* Shank1* mutations in the pathology of autism.* Shank1*-mutant mice displayed functional alterations in synaptic transmission as a consequence of a modified protein composition and morphology of the PSD and a reduced size of dendritic spines [175]. These observations resulted in an altered cognitive and communicative function [176]. Mutant mice exhibited an enhanced anxiety behavior, a decreased long-term memory, and a deficit in motor coordination. Surprisingly, although a decrease in the long-term retention of information was observed,* Shank1*-mutant mice were able to improve their spatial learning [175, 176, 264]. Furthermore, a promoter variant in the* Shank1* gene has been related to the symptoms of patients with SCZ [177] as well as a de novo Shank1 mutation [178].

Several human genetic studies have indicated that Shank2 is involved in ASD and ID [179, 180, 183], and two simultaneous studies produced a* Shank2* null mouse model to understand the molecular mechanisms underlying this pathology [181, 182].* Shank2* null mice presented fewer dendritic spines and reduced basal synaptic transmission [181]. The behavior of the animals was similar to ASD, with abnormalities in vocal and social behavior [181, 182]. Furthermore, Shank null mice showed reduced NMDAR functions, and the rescue of this mutation resulted in significantly improved animal social behavior [182]. The latter finding suggests that NMDAR function could be a target for patients with ASD. However, care must be taken with this approach because different levels of glutamate receptor expression are observed between* Shank2*(−/−) and* Shank3αβ*(−/−) mutants [181], suggesting that any treatment should consider the synaptopathic phenotype.

*Shank2* rare variants have also been identified in SCZ patients [185], and one of these variants has been related to ID [184]. Therefore, several lines of evidence indicate that Shank2 protein is a potential therapeutic target for autism and SCZ synaptopathologies.

Loss of one copy of the* Shank3* gene (haploinsufficiency) is considered the most prevalent monogenic cause of ASD [186]. Moreover, ASD is characterized by de novo mutations and deletions in* Shank3*, while truncating mutations are typically observed in ID patients [187]. In addition to its involvement in ASD, SCZ, and ID, Shank3 is the main protein responsible for the neuropsychiatric symptoms that occur in PMS patients [194], which manifest common characteristics of ID to varying degrees, delayed or absent speech, ASD-related symptoms, motor delays, and epilepsy. However, the clinical features of PMS are highly variable depending on the corresponding mutation. Translocations [195], de novo or truncating mutations [196], are* Shank3* mutations associated with PMS. The* Shank3* mutations, R1117X and R536W, have been observed in patients with SCZ associated with ID [197]. To understand the function of Shank3, mutant mouse models were generated; two different mutant mice carrying a deletion of exons 4–9 (JAX 017890 [188] and JAX 017442 [189]) showed repetitive behavior and impaired motor performance and cognitive dysfunction. Other animal models of Shank3 mutations include a deletion of exons 4–7,* Shank3 (4–7)*, and a deletion of exons 13–16,* Shank3 (13–16),* in which the mutant mice manifest anxiety and altered social and repetitive behaviors [190]. Deletion of exon 21 in Shank3 (21) [191] produced some ASD-related behaviors in mice together with impaired learning and memory, and a mouse model with an exon 9 deletion,* Shank3 (9)*, manifested mildly impaired spatial memory [192].

Shank3 is a well-known PSD protein in excitatory synapses and plays an important role in synaptic plasticity and functional coupling between presynaptic neurotransmitter release and a precise and rapid postsynaptic response. However, less is known about how defects in Shank3 could participate in the pathology of diseases such as ASD and SCZ. A recent study showed that Shank3 activation and localization in rat hippocampal neuron dendritic spines is regulated by zinc [193]. Interestingly a* Shank3* mutation (*Shank3* (R87C)) found in some ASD patients retains its zinc sensitivity but does not regulate the reliability of presynaptic neurotransmitter release. The latter does not preclude the possibility that other* Shank3* mutants or the other zinc-sensitive isoforms of* Shank2* interact abnormally with zinc, a metal that participates in synaptic plasticity and exhibits homeostatic dysregulation in ASD and other neurological disorders [265, 266].

#### 3.3.3. Homer

Homer is a family of scaffolding proteins formed by three members with a conserved aminoterminal enabled/vasodilator-stimulated phosphoproteins homolog 1 (EVH1) domain that binds to proline-rich sequences of mGluR [267, 268], inositol 1,4,5-triphosphate receptor (IP_3_R) [269], ryanodine receptors [270], transient receptor potential canonical-1 (TRPC1) ion channels [271], and Shank [272], functioning as adaptor proteins for several postsynaptic proteins [273]. The* Homer 1* gene undergoes alternative splicing to produce two isoforms, a short cytosolic version called Homer 1a, the expression of which increases after neuronal activation, mainly in frontal-subcortical neuronal circuits that are associated with neuropsychiatric disorders [274]. The long versions, Homer 1b/c, which are constitutively expressed and localized at the PSD, can form dimers through their carboxy-terminal domain [273]. These dimers bind to Shank and the metabotropic glutamate receptors mGluR1 and mGluR5, as well as indirectly to NMDAR and AMPAR through Shank. Therefore, Homer 1b/c form large protein complexes at the PSD, allowing the interaction of PSD proteins and signaling pathways. The short isoform, Homer 1a, acts as a dominant-negative of the long Homer isoforms, regulating the interactions of Homer with PSD proteins and suggesting that Homer 1 is a key organizer of the PSD, regulating the function of postsynaptic receptors and synaptic spine morphogenesis [259]. A stress condition that induces an increase in the expression of the Homer short isoform in rat limbo-corticostriatal structures [275] and hippocampus [276] suggests that this isoform regulates the consolidation of memories of stressful situations. Accordingly,* Homer 1* KO mice exhibit an exacerbated behavioral response to stressors because, in the absence of Homer 1, there is no buffering response to anxiety [198]. The latter could explain the mechanism by which the absence of Homer 1 triggers depression in humans.* Homer 1* KO mice also have behavioral and neurochemical abnormalities with a SCZ-like phenotype. Rescue experiments in those animals with Homer 1a and Homer 1c suggest different roles for this proteins in behavioral responses after stress [198]. In humans, linkage studies have identified susceptibility to SCZ in chromosomal loci containing the Homer 1 gene [199–201]. Moreover, a role for Homer 1 in SCZ in humans was validated in a recent study in which an association was found between two Homer 1 polymorphisms and the Positive and Negative Syndrome Scale (PANSS), a medical scale used for measuring the symptom severity of patients with SCZ [202]. Taken together, defects in Homer proteins will produce alterations in the architecture and function of the PSD, ultimately resulting in a neurological disease.

#### 3.3.4. SynGAP1

SynGAP1, which is a postsynaptic component of the PSD, plays an important and essential role in the development of cognition and proper synaptic function. It has been reported that SynGAP interacts with PSD-95 [248] and is phosphorylated by Ca^2+^/calmodulin-dependent protein kinase II (CamKII), which in turn is activated by increased Ca^2+^ levels induced by NMDAR activation [277]. This phenomenon suggests that SynGAP develops an essential role in the NMDAR-dependent activation of Ras signaling pathways and in synaptic plasticity.

Mice with a heterozygous null mutation of* SynGAP* manifest impaired learning and memory associated with decreased synaptic transmission and NMDAR-mediated synaptic currents [278]. Moreover, SynGAP function has been studied in a mouse model of SCZ. As explained above, NMDARs are involved in the pathophysiology of SCZ, and it has been reported that reduced expression of SynGAP also results in abnormal behaviors, as demonstrated by abnormal functions of NMDARs such as a persistent hyperactivity and social and working memory loss, among others [203]. In addition to interacting with the aforementioned proteins and receptors, SynGAP is associated with other postsynaptic proteins such as PSD-95, the synapse-associated protein-102 (SAP-102), and PSD-93, NRXNs, and NLs, proteins that have been related to ASD [204]. The excitatory and inhibitory synaptic balance is altered in patients with ASD [279], and it has also been reported that SynGAP may regulate this synaptic balance in cortical neurons [205]. Furthermore, a recent study established a relationship between SynGAP and ID. Patients with a de novo mutation, haploinsufficiency for* SynGAP1*, were found to manifest epilepsy, hypotonia, constipation and other ID-associated symptoms [206].

#### 3.3.5. Gephyrin

Although in this review we preferentially considered the most important pre- and postsynaptic proteins involved in synaptopathies located at the AZ and PSD, respectively, of excitatory synapses, gephyrin (gphn) is also included because it is a key scaffolding protein at the postsynaptic membrane that plays an essential role through its interactions with NL2, a previously described protein, and collybistin in the clustering and localization of glycine and the *α* and *β* subunits of GABA_A_ receptors at inhibitory synapses [220]. It has been reported that* gphn* KO mice, however, display correct glutamate receptor localization; they manifest a loss of postsynaptic GABA_A_ and glycine receptor clustering [280].

Interestingly, gphn is well-known to be involved in several neurological disorders including ASD, SCZ, and epilepsy because it is functionally linked to various synaptic proteins that represent a genetic risk for the development of neurological diseases such as NLs, NRXNs, and collybistin. In particular, exonic gene microdeletions in* gphn*, including hemizygous microdeletions, de novo, and paternally inherited deletions have been found in different ASD families. They displayed ASD-related symptoms such as motor and social impairment, repetitive and impulsive behaviors, anxiety, and obsessive-compulsive disorders [207]. A recent study has reported a novel exonic* gphn* microdeletion in patients with idiopathic generalized epilepsy, confirming that genetic alterations constitute a risk factor for neuropsychiatric disorders through the impairment of GABAergic inhibitory synaptic transmission [208].

### 3.4. Other Postsynaptic-Associated Proteins 

#### 3.4.1. Disrupted in Schizophrenia 1, DISC1

DISC1, a protein encoded by the* DISC* gene, has been found in asymmetric synapses, principally on the postsynaptic side [281]; in fact, a bioinformatics analysis of DISC1 interactions suggested that DISC1 is an essential component of the PSD and a key player in the regulation of synaptic plasticity [282]. The DISC1 gen locus has been considered a risk factor because the (1;11)(q42;q14.3) translocation was observed in different members of a Scottish family that manifested clinical phenotypes associated with BPD, SCZ, and depression [209, 210]. Moreover, a DISC1 intragenic microsatellite has been associated with autism, whereas a SNP of DISC1 has been related to Asperger syndrome in Finnish families [214].

Different mouse models have reported that DISC1 is involved in synapse function and related to neurological diseases such as depression [213]. It is known that C57BL/6J mice carry an exonic deletion in* Disc1*, which produces a truncated DISC1 protein that mimics the putative phenotypic effects of the disease-associated chromosomal translocation, resulting in memory impairment and fewer synaptic spines [211]. In contrast, the downregulation of DISC1 by shRNA in adult C57BL/6 mice elicited an accelerated dendritic development and synapse formation in both GABAergic and glutamatergic synapses of newborn neurons [283]. As previously described, SCZ is a neurological disorder characterized by disrupted synaptic connectivity. Likewise, prolonged knockdown of DISC1 has been shown to induce synaptic deterioration, and inhibition of the signaling pathways triggered by DISC1 improves the behavioral deficit manifested in the DISC1 knockdown mouse model [284].

In addition, it is noteworthy that, surprisingly, an increased amount of insoluble DISC1 oligomer aggregates was detected in the postmortem brain of SCZ patients, demonstrating a common link with other neurological disorders characterized by protein aggregation such as AD and HD [212]. A novel study has reported significantly enhanced levels of APP fragments, as well as decreased levels of A*β*_42_ and A*β*_40_ as a consequence of DISC1 knockdown, suggesting that DISC1 participates in the proteolytic processing of APP and thus establishing a relationship with the pathology of AD [215].

## 4. Piccolo and RIM, an Example of Multidomain Presynaptic Proteins with Diverse Functions

**Figure 4:**
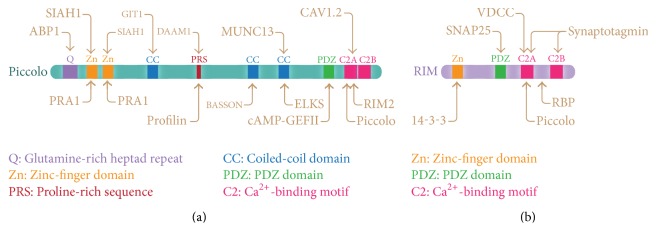
Domain structure of two active zone proteins associated with synaptopathies. The diagrams show the multimodular organization of (a) Piccolo and (b) RIM, and their interaction with other proteins. Arrows indicate binding reactions. Domains are shown in colored boxes and designations are indicated by standard abbreviations.

A highlight characteristic of several AZ proteins, such as Piccolo and RIM, is their multidomain structure. Piccolo and RIM form homo and hetero-oligomers at the CAZ and exert their synaptic functions through molecular interactions with different binding targets. The functions and interactions of Piccolo include (a) actin cytoskeleton dynamic (profilin, Daam1, Abp1, and GIT1), (b) exocytosis (cAMP-GEFII), (c) endocytosis (PRA1 and GIT1), (d) protein turnover (Siah1), (e) membrane trafficking (Epc2), (f) calcium signaling (L-type Ca^2+^ channel), (g) scaffolding (Bassoon, RIM, Munc13, and ELKS), and (h) SV priming (RIM, Munc13) (Figure 4(a)). RIM has prominent roles in (a) SV docking and priming (SNAP25, synaptotagmin), (b) scaffolding (Piccolo, ELKS, 14-3-3), and (c) calcium channel signaling (RBP, N- and P/Q-type Ca^2+^-channels) (Figure 4(b)).

Therefore, mutations on Piccolo and/or RIM genes rendering a mutated protein or altered levels of protein expression could produce functional imbalances at the synapse due to abnormal interactions with their respective partners. Consequently, neuronal circuits might be impaired responding suboptimally to environmental requirements affecting synaptic plasticity, which is known to be altered in many neurological diseases.

## 5. Concluding Remarks

Genetic studies of human synaptopathies together with animal models have revealed that in most cases a disease cannot be explained by the gene mutation of a single synaptic protein, and, similarly, abnormal individual expression of different synaptic proteins can trigger the same or a similar disease phenotype. Accordingly, GWA studies that have identified specific genes associated with synaptopathies sometimes do not replicate all of the symptomatology of the disease in animal models, suggesting the participation of other genes. This phenomenon is not unexpected because synaptic proteins are coupled to a highly dynamic interactome that regulates basal and plastic synapse functions. Hence, additional GWA studies are necessary to identify most of the defective gene variants and the brain region harboring the molecular alteration in a specific synaptopathy. Such findings in humans may be used to create suitable animal models that closely mimic the human defect, allowing detailed studies of the physiological alterations. Therefore, this will allow the consideration of specific pharmacological therapies for the underlying synaptopathic genotype and phenotype.

## Figures and Tables

**Table 1:** Presynaptic proteins involved in different synaptopathies and their role in physiological synaptic function.

	Function	Neurological disease	References
*Synaptic vesicles proteins*			

Synapsin 1	Mobilization, release, and tethering of SV to the cytoskeleton away from the AZ	BPD	[23]
		Epilepsy	[24, 25]
		ASD	[25]

Synapsin 2	SVs mobilization and regulation of the number and density of the reserve pool	SCZ	[23, 26–30]
		Epilepsy	[31]
		BPD	[23, 32]

Synapsin 3	Synaptogenesis and modulation of neurotransmitter release	SCZ	[23, 33]
		BPD	[23]

Synaptophysin	Control of SVs endocytosis	SCZ	[34]
		BPD	[35]

*Cytomatrix of active zone proteins*			

RIMs	Docking, SV fusion, and neurotransmitter release Synaptic plasticity	SCZ ASD	[36, 37] [38, 39]

Piccolo	AZ scaffolding protein	SCZ	[37]
		MDD	[40–42]
		BPD	[43]

*SNARE proteins*			

SNAP25	Mediation of vesicle docking and fusion	BPD	[35, 44, 45]
		SCZ	[44, 46, 47]
		ADHD	[48–50]

*Adhesion molecules*			

SynCAM1	Synapse formation, synaptic plasticity, and axonal pathfinding	ASD	[51–54]

Cadherin	Selection of neuronal target, synapse formation, and plasticity	SCZ	[55, 56]
		BPD	[55]
		ASD	[55, 57–60]
		ADHD	[61]

NRXN1	Formation and maturation of the synapse	ASD	[62–65]
		SCZ	[66–68]

The table summarizes the physiological synaptic function of presynaptic proteins whose alterations result in synaptopathies related to neurodevelopmental, neuropsychiatric, and neurodegenerative diseases. ADHD, attention deficit hyperactivity disorder; ASD, autism spectrum disorder; AZ, active zone; BPD, bipolar disorder; MDD, major depressive disorder; NRXN, neurexin; RIM, Rab3a interacting molecule; SCZ, schizophrenia; SynCAMs, Synaptic adhesion molecules; SV, synaptic vesicle.

**Table 2:** Postsynaptic proteins involved in different synaptopathies and their role in physiological synaptic function.

Protein	Function	Neurological disease	References
*Adhesion molecules*			

NL1	Memory formation and maturation of excitatory synapses	ASD	[111, 112]
		AD	[113]
		FXS	[114]

NL2	Formation and remodeling of inhibitory synapses	SCZ	[115]
		ASD	[116]

NL3	Formation and remodeling of excitatory and inhibitory synapses	ASD	[105, 117–121]

NL4	Formation and remodeling of excitatory and inhibitory synapses	ASD	[117–119, 122–126]

*Glutamate receptors*			

NMDARs	Regulation of synaptic plasticity and memory formation	ASD	[127–129]
		SCZ	[127, 130, 131]
		AD	[132–136]
		HD	[137, 138]

KARs	Maturation of neural circuits during development	ASD	[139–141]
		SCZ	[142]
		BPD	[142, 143]

AMPARs	Mediators of excitatory transmission and synaptic plasticity	ASD	[144]
		SCZ	[145–147]
		BPD	[148]
		MDD	[149]
		FXS	[150, 151]
		HD	[152]

mGluRs	Regulation of neuronal excitability, learning, and memory	ASD	[153–156]
		ID	[156]
		FXS	[157–159]

*Scaffolding proteins*			

PSD-95	Stabilization of the synapse, and regulation of synaptic strength, transmission, and plasticity	AD	[160–162]
		ASD	[163, 164]
		SCZ	[164, 165]
		HD	[166–168]
		FXS	[169–173]

Shank1	Regulation of the structural and functional organization of the dendritic spines	ASD	[174–176]
		SCZ	[177, 178]

Shank2	Synaptogenesis; regulation of the molecular structure and modulation of interacting proteins in the PSD	ASD	[179–182]
		ID	[183, 184]
		SCZ	[185]

Shank3	Synapse formation, dendritic spine maturation, and synaptic plasticity	ASD	[186–193]
		PMS	[194–196]
		SCZ	[197]

Homer	Organization, stabilization and function of the PSD, and contribution in dendritic spine morphogenesis	SCZ	[198–202]

SynGAP	Involvement in the cognitive development and synaptic transmission and function	SCZ	[203]
		ASD	[204, 205]
		ID	[206]

Gephyrin	Clustering and localization of glycine and GABA receptors at inhibitory synapses	ASD	[207]
		*SCZ*	
		Epilepsy	[208]

*Other postsynaptic-associated proteins*			

DISC1	Regulation of synaptic plasticity	SCZ	[209–212]
		Depression	[209, 213]
		BPD	[210]
		ASD	[214]
		AD	[215]

The table summarizes the physiological synaptic function of postsynaptic proteins whose alterations result in synaptopathies related to neurodevelopmental, neuropsychiatric, and neurodegenerative diseases. AD, Alzheimer's disease; AMPARs, *α*-amino-3-hydroxy-5-methyl-4-isoxazolepropionic acid receptor; BPD, bipolar spectrum disorder; ASD, autism spectrum disorder; DISC1, disrupted in schizophrenia 1; FXS, Fragile X syndrome; HD, Huntington's Disease; ID, intellectual disability; KARs, kainate receptors; MDD, major depressive disorder; mGluRs, metabotropic glutamate receptors; NLs, neuroligins; NMDARs, *N*-methyl-D-aspartate; PMS, Phelan-McDermid syndrome; PSD-95, postsynaptic density-95; SCZ, schizophrenia.

## Abbreviations

A*β*: Amyloid-*β*
Abp1: Actin binding protein
AD: Alzheimer's disease
ADHD: Attention deficit hyperactivity disorder
AMPA: *α*-Amino-3-hydroxy-5-methyl-4-isoxazolepropionic acid
APP: Amyloid precursor protein
ASD: Autism spectrum disorders
AZ: Active zone
BPD: Bipolar disorder
CAZ: Cytomatrix at the active zone
CNS: Central nervous system
EVH1: Enabled/vasodilator-stimulated phosphoproteins homolog 1
F-actin: Filamentous actin
FMRP: Fragile X retardation protein
FXS: Fragile X syndrome
GABA: Gamma aminobutyric acid
gephyrin: gphn
GWA: Genome-wide association
HD: Huntington's Disease
Htt: Huntingtin
ID: Intellectual disability
iGluRs: Ionotropic-glutamate receptors
KARs: Kainate receptors
KO: Knock-out
LTP: Long-term potentiation
MDD: Major depressive disorder
NLs: Neuroligins
mGluRs: Metabotropic glutamate receptors
NMDA: * N*-Methyl-D-aspartate
NRXN: Neurexin
PANSS: Positive and negative syndrome scale
PMS: Phelan-McDermid syndrome
PKC: Protein kinase C
PRA1: Prenylated rab3A acceptor
PSD: Postsynaptic density
RIM: Rab3 interacting molecules
RIM-BP: RIM-binding protein
SCZ: Schizophrenia
shRNAi: Short hairpin RNA interference
SNAP25: Synaptosomal-associated protein 25
SNARE: SNAP Soluble NSF Attachment Protein Receptor
SNP: Single nucleotide polymorphism
SynCAMs: Synaptic adhesion molecules
SV: Synaptic vesicles
TrkB: Tropomyosin receptor kinase B
TRPC1: Transient receptor potential canonical-1
VDCCs: Voltage-dependent Ca^2+^ channels.
